# Genome-wide Parallelism Underlies Rapid Freshwater Adaptation Fueled by Standing Genetic Variation in a Wild Fish

**DOI:** 10.1093/molbev/msaf160

**Published:** 2025-07-03

**Authors:** Hao Yang, Yu-Long Li, Teng-Fei Xing, Jian-Hui Wu, Ting Wang, Ming-Sheng Zhu, Jin-Xian Liu

**Affiliations:** Key Laboratory of Marine Ecology and Environmental Sciences, Institute of Oceanology, Chinese Academy of Sciences, Qingdao 266071, China; Laboratory for Marine Ecology and Environmental Science, Qingdao Marine Science and Technology Center, Qingdao 266237, China; University of Chinese Academy of Sciences, Beijing 100049, China; Key Laboratory of Marine Ecology and Environmental Sciences, Institute of Oceanology, Chinese Academy of Sciences, Qingdao 266071, China; Laboratory for Marine Ecology and Environmental Science, Qingdao Marine Science and Technology Center, Qingdao 266237, China; Key Laboratory of Marine Ecology and Environmental Sciences, Institute of Oceanology, Chinese Academy of Sciences, Qingdao 266071, China; Laboratory for Marine Ecology and Environmental Science, Qingdao Marine Science and Technology Center, Qingdao 266237, China; Shanghai Aquatic Wildlife Conservation and Research Center, Shanghai 200092, China; Shanghai Monitoring Station of Aquatic Biological Resources in the Yangtze River Basin, Shanghai 202162, China; Shanghai Aquatic Wildlife Conservation and Research Center, Shanghai 200092, China; Shanghai Monitoring Station of Aquatic Biological Resources in the Yangtze River Basin, Shanghai 202162, China; Office of Taihu Fishery Management Committee, Suzhou 215104, China; Key Laboratory of Marine Ecology and Environmental Sciences, Institute of Oceanology, Chinese Academy of Sciences, Qingdao 266071, China; Laboratory for Marine Ecology and Environmental Science, Qingdao Marine Science and Technology Center, Qingdao 266237, China

**Keywords:** parallel evolution, rapid adaptation, complex traits, polygenic adaptation, standing genetic variation, allele frequency changes

## Abstract

A fundamental focus of ecological and evolutionary biology is determining how natural populations adapt to environmental changes. Rapid parallel phenotypic evolution can be leveraged to uncover the genetics of adaptation. Using population genomic approaches, we investigated the genetic architecture underlying rapid parallel freshwater adaptation of *Neosalanx brevirostris* by comparing four freshwater-resident populations with their common ancestral anadromous population. We demonstrated that the rapid parallel adaptation to freshwater followed a complex polygenic architecture and was characterized by genomic-level parallelism, which proceeded predominantly through repeated selection on the preexisting standing genetic variations. Frequencies of the genome-wide adaptive standing variations were moderate in the ancestral anadromous population, which had pre-adapted to fluctuating salinities. Relatively large allele frequency shifts were observed at some adaptive single-nucleotide polymorphisms (SNPs) during parallel adaptation to freshwater environments, with a large fraction of freshwater-favored alleles being fixed or nearly fixed. These adaptive SNPs were involved in multiple biological functions associated with osmoregulation, immunoregulation, locomotion, metabolism, etc., which were highly consistent with the polygenic architecture of adaptive divergence between the two ecotypes involving multiple complex physiological and behavioral traits. This work provides insight into the mechanisms by which natural populations rapidly evolve to changes in the environment and highlights the importance of standing genetic variation for the evolutionary potential of populations facing global environmental changes.

## Introduction

Identifying the genetic mechanisms driving adaptation is fundamental in ecological and evolutionary biology. Although biological phenotypes can evolve rapidly in response to environmental changes (Zong et al. 2021; McCulloch and Waters 2023), the genomic underpinnings of such rapid evolution remain elusive. Classical population genetics provides evidence for adaptation through selective sweeps, leading to dramatic allele frequency changes at single or a few loci (Panigrahi et al. 2023). Conversely, quantitative genetics presumes that adaptation underlain by highly polygenic traits can proceed via subtle allele frequency shifts across numerous loci (Pritchard and Di Rienzo 2010). The polygenic model poses distinct challenges: the weak effects of abundant contributing loci often fall below the detection limits of outlier detection methods, as they become statistically indistinguishable from background noise generated by genetic drift and sampling biases (Yeaman 2015; Buffalo and Coop 2020). On the other hand, polygenic adaptation may be underlain by multiple traits, and some of which might have simpler genomic architectures and could be independently targeted by strong selection (Höllinger et al. 2019).

Parallel evolution systems where convergent phenotypes have evolved repeatedly across independent populations can help to reveal the genetic basis of adaptive evolution (Moran et al. 2023). Evolutionary genomic studies increasingly utilize multiple replicated populations to detect selected loci with large effects (Han et al. 2020; Winchell et al. 2023) and identify sources of adaptive genetic variations (Konecná et al. 2021). Meanwhile, allele frequency covariation across replicated genomic data reveals promising additional signals, as alleles associated with specific fitness backgrounds are expected to change frequencies in consistent directions when selection pressures are similar (Buffalo and Coop 2020; Reid et al. 2023). Previous studies have elucidated that repeated adaptive evolution occurs mainly through three processes, including selection on standing variation in the ancestral population, transporting adaptive alleles via gene flow among populations, or de novo mutations occurring at the same locus (Alves et al. 2019; Tan et al. 2020; Louis et al. 2021; Waters and McCulloch 2021). Among them, the preexistence of adaptive alleles in the ancestral population can significantly enhance both the probability of parallel evolution and the speed of adaptation (Barrett and Schluter 2008; Ralph and Coop 2015). On the other hand, several factors such as genetic redundancy (Barghi et al. 2019; Barghi et al. 2020), demographic dynamics (Bailey et al. 2017), and population-specific environmental heterogeneity (Stuart et al. 2017) can lead to non-parallel adaptive signatures.

Evolutionary transitions from marine to freshwater habitats have played an essential role in generating phyletic diversity within fishes (Vega and Wiens 2012). It is hypothesized that estuarine and anadromous species, with their inherent capacity to tolerate varying salinities, may be particularly predisposed to such ecological shifts (Brennan et al. 2018). Transitions from marine to freshwater represent dramatic changes between “adaptive zones”, where many environmental and ecological features vary (e.g. salinity, dissolved oxygen, pH, food, predators, pathogens, etc.; Lee and Bell 1999). Therefore, successful freshwater colonization typically requires coordinated adaptation across multiple polygenic traits, including osmoregulation, immunoregulation, locomotion, and metabolism (Brennan et al. 2018). Genomic studies have revealed diverse adaptive pathways underlying repeated colonization from marine to freshwater. Some species exhibited parallel evolutionary trajectories via standing genetic variation, either distributed across the genome (Jones et al. 2012) or localized in regions of chromosomal structural variation (Zong et al. 2021). Others evolved through non-parallel solutions involving divergent mutations in homologous genes or substitutions in different genes (Perrier et al. 2013). Generally, the degree of parallelism in adaptive evolution varies across biological hierarchies, being highest at the phenotypic level, intermediate at the pathway level, and lowest at the genomic level (Schloetterer 2023). A critical determinant of genomic parallelism is the nature of founder populations. Experimental evolution studies in *Drosophila* have demonstrated that populations derived from a single ancestral source exhibit significantly greater parallelism in genomic responses than those originating from multiple, genetically distinct founder populations (Schloetterer 2023).

The short-snout icefish (*Neosalanx brevirostris*) is an annual fish exhibiting two different ecotypes. One is an anadromous ecotype, widely distributed along the coasts and estuaries of China (Zhang 1987; Guo et al. 2011), where immature fish grow before migrating to freshwater rivers or lakes for spawning. After reproduction, the adults die and the newly hatched juveniles migrate back to coastal habitats. The other is a freshwater-resident ecotype spending its entire life cycle in the landlocked rivers and lakes of the middle and lower reaches affiliated with the Yangtze River and Huaihe River (Zhang 1987; Guo et al. 2011). Between the mid-1950s and 1980s, the construction of massive water conservancy projects landlocked some anadromous individuals in freshwater lakes (Zhu et al. 2007; Lin et al. 2013; Liang et al. 2022), where they rapidly adapted and the freshwater-resident ecotype evolved, which gradually became the dominant species in these lake ecosystems (Mao et al. 2011; Liang et al. 2022). Phylogeographic analysis using mitochondrial cytochrome b (mt-*cytb*) sequences revealed no discernible genetic differentiation between the anadromous and freshwater-resident populations (Zhao et al. 2008), suggesting a close genetic relationship between the two ecotypes. Coalescent simulations, also utilizing mt-*cytb* sequences, further demonstrated that different freshwater-resident populations derived from the anadromous population through multiple unrelated long-distance dispersal events in the Yangtze River basin (Zhao et al. 2011). These findings suggest that the anadromous population and the freshwater-resident populations in the Yangtze River basin may represent a system of independent parallel freshwater adaptation. Thus, this natural system provides a compelling opportunity to infer the genetic mechanisms underlying rapid adaptive evolution to environmental change that involves multiple complex polygenic traits.

Using population genomic approaches, we aim to elucidate the genomic architecture underlying rapid parallel adaptation to freshwater environments in *N. brevirostris* by comparing four freshwater-resident populations with their ancestral anadromous population in the Yangtze River system. Our study specifically focuses on two core questions: (i) What is the primary source of genetic variation that contributes the most to parallel freshwater adaptation, standing variations, or de novo mutations? and (ii) To what extent are parallel phenotypic adaptations reflected at the genomic level? To resolve these questions, we first characterized the genomic parameters, including genetic diversity, population structure, and demographic dynamics. We then implemented genome-wide scans to identify adaptive variants associated with polygenic freshwater adaptation. These results will provide a general understanding of the genetic basis of rapid adaptation of complex traits in wild populations driven by environmental changes.

## Results

### Genetic Diversity and Population Genetic Structure

**Fig. 1.**
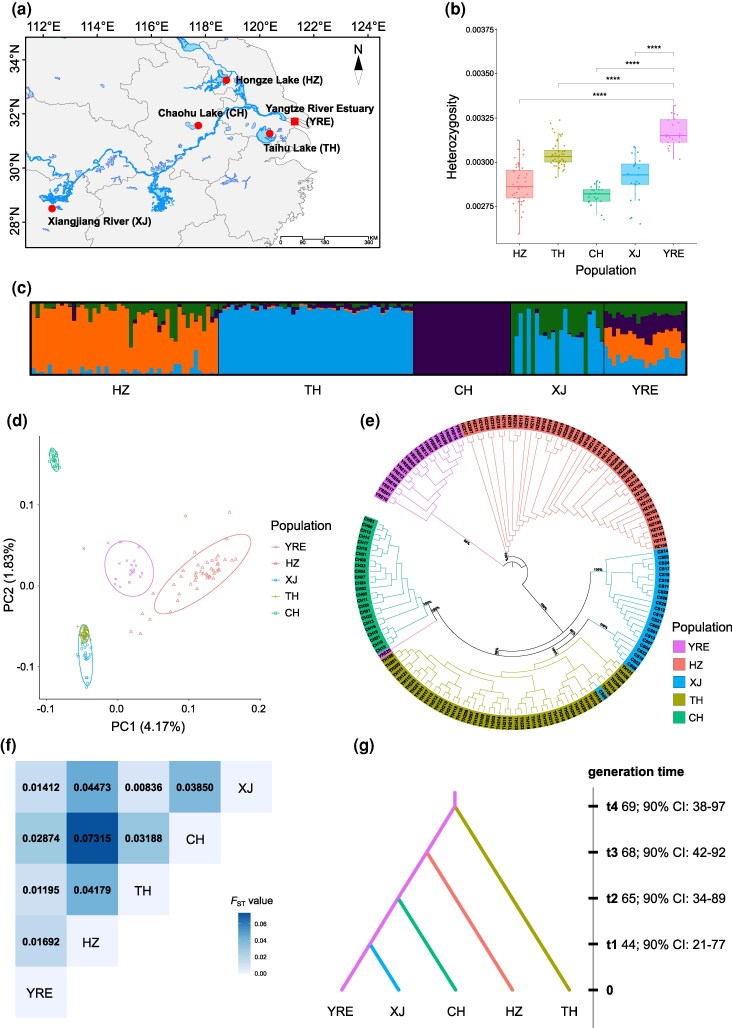
Sampling map, genetic diversity, population genetic structure, and evolutionary history of *N. brevirostris* populations. a) Sampling locations for the five populations, one anadromous population from the Yangtze River Estuary (YRE) and four freshwater-resident populations from Hongze Lake (HZ), Taihu Lake (TH), Chaohu Lake (CH), and Xiangjiang River (XJ). b) Distribution of individual heterozygosity for five populations. Boxplot elements: center line = median, box limits = 25th and 75th percentiles. Significance: ****P < 0.0001 (Wilcoxon rank sum test). c) Population structure inferred by Admixture analysis at *K* = 4. d) PCA showing the clustering of individuals along the first two PCs, with color and shape labels representing different populations. e) An individual-level neighbor-joining tree with key nodes support values (Branch length information can be found in the supplementary fig. S3, Supplementary Material online). f) Pairwise *F*_ST_ values among the five populations, with significant values after Bonferroni correction highlighted in bold. g) The most probable evolutionary scenario with divergence times estimated by DIYABC-RF.

Whole genome resequencing of 161 *N. brevirostris* individuals (supplementary fig. S1, Supplementary Material online) from five populations was performed with an average depth of 29× (ranging from 19× to 46×; Fig. 1a and supplementary table S1, Supplementary Material online). After aligning the resequencing data to the reference genome, a total of 13,823,324 variable sites were scored. Filtering produced a dataset of 2,036,542 high-quality biallelic single-nucleotide polymorphisms (SNPs) distributed across 28 chromosomes of the reference genome (supplementary fig. S2, Supplementary Material online).

The levels of individual heterozygosity were comparable across five populations, with median values ranging from 0.00283 to 0.00316 (Fig. 1b and supplementary table S1, Supplementary Material online). As anticipated, individual heterozygosity in the ancestral anadromous Yangtze River Estuary population was significantly higher than that in the four freshwater-resident populations (Wilcoxon rank sum test, *P* < 2.2e-16).

The *K* = 4 was chosen as the optimal *K* in Admixture analysis, revealing that genetic variation was partitioned by geography (Fig. 1c). Notably, the anadromous Yangtze River Estuary population contained all genetic components present in the four freshwater-resident populations, consistent with its ancestral status in this system. Principal component analysis (PCA) based on the first two PCs clearly separated the Yangtze River Estuary, Hongze Lake, and Chaohu Lake populations, while several individuals from the Xiangjiang River overlapped with those from Taihu Lake (Fig. 1d). This pattern was consistent with the individual-based neighbor-joining (NJ) tree (Fig. 1e and supplementary fig. S3, Supplementary Material online). However, both Admixture results at *K* = 5 or 6 (supplementary fig. S4, Supplementary Material online) and PC1-PC3/PC2-PC3 plots (supplementary fig. S5, Supplementary Material online) consistently supported that the Taihu Lake and Xiangjiang River populations are genetically distinct from each another. Moreover, the genome-wide fixation indexes (*F*_ST_) of all freshwater-anadromous ecotype pairs were generally low but statistically significant, ranging from 0.01195 (Yangtze River Estuary vs. Taihu Lake) to 0.02874 (Yangtze River Estuary vs. Chaohu Lake), indicating that the freshwater-resident populations were recently derived from their anadromous ancestor (Fig. 1f).

### Evolutionary History and Recent Demographic Dynamics

The most probable evolutionary scenario, receiving the highest classification votes in DIYABC-RF analysis (supplementary table S2, Supplementary Material online), was depicted in Fig. 1g. According to this scenario, the Taihu Lake population derived first from the Yangtze River Estuary population approximately 69 (90% CI: 38 to 97) generations ago, followed by sequential formation of the Hongze Lake, Chaohu Lake, and Xiangjiang River populations at about 68 (90% CI: 42 to 92), 65 (90% CI: 34 to 89), and 44 (90% CI: 21 to 77) generations ago, respectively.

Consistent with the population history outlined in the introduction, GONE analysis of recent demographic dynamics based on the linkage disequilibrium (LD) method revealed two key patterns (supplementary fig. S6, Supplementary Material online): (i) all freshwater-resident populations except for Hongze Lake showed signals of recent demographic expansions, and (ii) the effective population sizes (*N*_e_) of Yangtze River Estuary population maintained stable, followed by a notable increase during the most recent past 20 generations.

Meanwhile, analyses of the genome-wide 8,614,644 biallelic SNPs (without minor allele frequency [MAF] filtering) revealed two consistent signatures across all five populations: (i) a pronounced L-shaped site frequency spectrum (SFS) with an excess of rare variants (75.7% to 79.6% of segregating sites with MAF ≤ 0.05; supplementary fig. S7, Supplementary Material online), and (ii) predominantly negative Tajima's *D* values in 100-kb sliding windows with zero step size (99.3% to 100% of windows showing *D* < 0, with means ranging from −1.385 to −0.705; supplementary table S3, Supplementary Material online). These genomic signatures indicated that none of the populations had undergone strong genetic drift and provided evidence for recent demographic expansions.

### Candidate Outlier SNPs and Parallel Allele Frequency Shifts

**Fig. 2.**
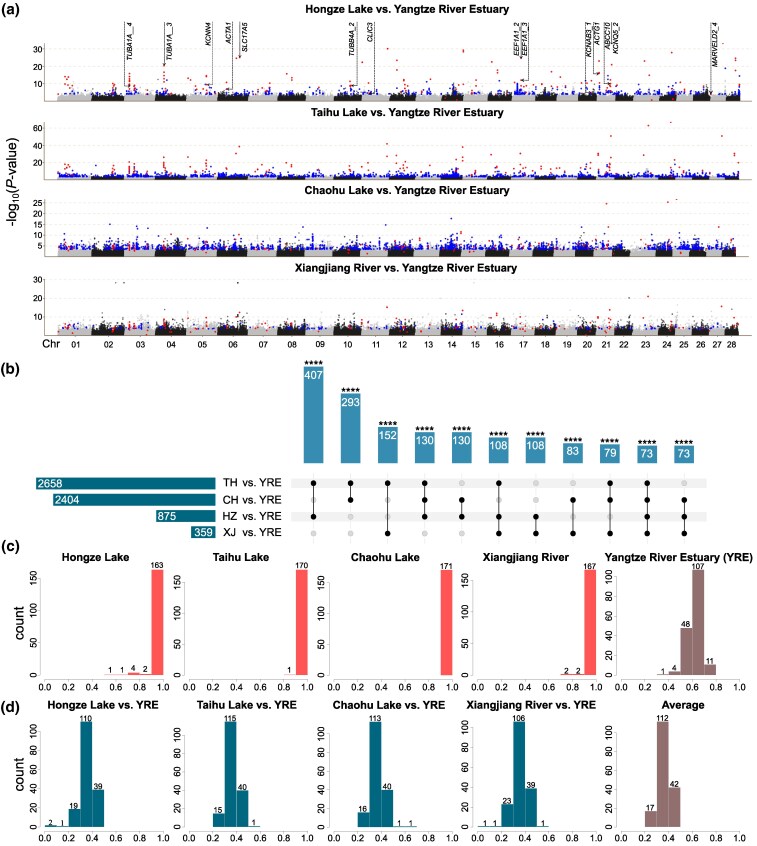
Candidate outlier SNPs and parallel allele frequency shifts. a) Manhattan plot showing the distribution of outlier SNPs for the four freshwater-anadromous ecotype pairs. *Y*-axis represents *P*-values calculated by pcadapt on -log_10_ scale. Blue dots represent the outliers detected by both the FET and pcadapt methods in each ecotype pair. Red dots represent the outliers detected by both FET and pcadapt methods and shared among three or four ecotype pairs. Some important genes with their corresponding outliers are labeled. b) Upset plot displaying the number of overlapping outliers in all combinations of the four ecotype pairs. Significance: ****P < 0.0001 (hypergeometric test). c) Frequency distribution of the freshwater-favored allele (FWA) for the 171 candidate outlier SNPs in the five populations. d) Frequency increment distribution of the FWA for the 171 candidate outlier SNPs in the four ecotype pairs and their means. CH, Chaohu Lake; HZ, Hongze Lake; TH, Taihu Lake; XJ, Xiangjiang River; YRE, Yangtze River Estuary.

Using both Fisher's exact test (FET, FDR < 0.05) and pcadapt (*Q*-value < 0.1), we identified a total of 124,698 outlier SNPs with significantly elevated genetic differentiation compared to neutral expectations, potentially linked to adaptive divergence between the freshwater-resident and anadromous ecotypes across all four freshwater-anadromous ecotype pairs. Of these, FET and pcadapt identified 113,064 and 17,659 outliers, respectively, with 5,440 outliers detected by both methods among the four ecotype pairs (supplementary table S4, Supplementary Material online). The number of shared outlier SNPs detected by both methods varied among ecotype pairs, ranging from 359 in Xiangjiang River versus Yangtze River Estuary to 2,658 in Taihu Lake versus Yangtze River Estuary (Fig. 2a). At last, we identified 171 outlier SNPs detected by two methods and shared across at least three ecotype pairs (Fig. 2a), which were distributed across 28 chromosomes, with 1 to 17 SNPs on each (supplementary fig. S8a, Supplementary Material online). Although outlier detection is agnostic of directionality in each ecotype pair, consistency in the direction of allele frequency change was observed for all 171 outlier SNPs across all four freshwater-anadromous ecotype pairs. Further analysis found that the overlap of the shared outlier SNPs across all combinations of the four ecotype pairs showed significant parallelism (non-random number of overlapping outliers; hypergeometric test, *P* < 0.0001; Fig. 2b and supplementary table S5, Supplementary Material online). Therefore, these 171 SNPs represent high-confidence candidates for parallel freshwater adaptation.

We defined alleles showing increased frequency in all four freshwater-resident populations relative to the ancestral Yangtze River Estuary population as freshwater-favored alleles (FWAs). Relatively large allele frequency shifts were observed for the FWAs of all the 171 candidate outlier SNPs in all freshwater-anadromous ecotype pairs (Fig. 2c and d). The FWAs of most candidate outlier SNPs were either fixed or nearly fixed in all freshwater-resident populations, with an average frequency of 0.988 (Fig. 2c). In the ancestral Yangtze River Estuary population, the FWAs of all 171 candidate outlier SNPs occurred as standing genetic variations, exhibiting moderate frequencies with an average of 0.621. The frequency of the FWA of most candidate outlier SNPs (85% to 91%) had increased by at least 0.3 in the freshwater-resident populations, with average frequency increments ranging from 0.361 in Hongze Lake to 0.372 in Taihu Lake across all candidate outlier SNPs (Fig. 2d).

### Characteristics of Covariant SNPs

**Fig. 3.**
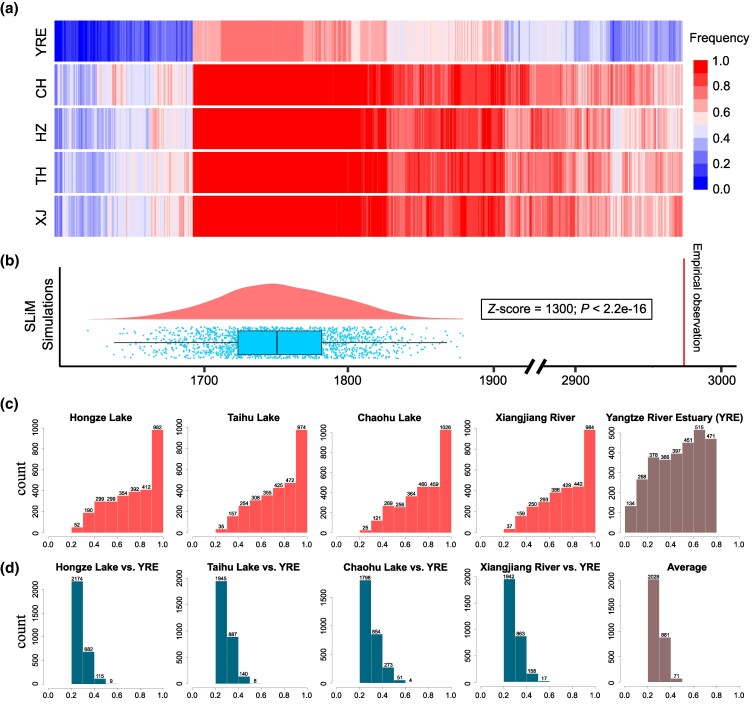
Characteristics of candidate covariant SNPs. a) Heatmap of the freshwater-favored allele (FWA) frequency for the 2,980 candidate covariant SNPs in the five populations. b) Distribution of the neutral expected number of covariant SNPs with a frequency increment (ΔFWA) ≥ 0.2 obtained through SLiM simulations and their statistical significance compared with the actual observed values. c) Frequency distribution of the FWA for the 2,980 candidate covariant SNPs in the five populations. d) Frequency increment distribution of the FWA for the 2,980 candidate covariant SNPs in the four ecotype pairs and their means. YRE, Yangtze River Estuary; CH, Chaohu Lake; HZ, Hongze Lake; TH, Taihu Lake; XJ, Xiangjiang River.

Given the limited sensitivity of outlier detection for small allele frequency shifts and the anticipated covariant allelic changes in parallel adaptation, we conducted allele frequency covariant analysis, to specifically identify covariant SNPs with one allele conforming to the FWA definition, in the expectation of observing more parallel genomic signals. Initially, we identified 604,923 covariant SNPs, which included 3,031 (56%) of the 5,440 outlier SNPs mentioned above, statistically more than the neutral expectation of 680 (12.5%; 0.5^4 × 2; χ² test, *P* < 2.2e-16) under an ideal scenario (no recent genetic drift, without recombination, and absence of sampling error). To minimize the effects of random genetic drift and sampling variance, we conservatively selected the dataset with FWA frequency increments≥ 0.2 for downstream analysis (Fig. 3a and supplementary table S6, Supplementary Material online). The final covariant dataset contained 2,980 SNPs, which was significantly larger than the expectations obtained from SLiM simulations performed under a neutral scenario (mean ± SD: 1,753 ± 42), considering the actual demographic history, recombination, and sampling variance (*Z*-test, *P* < 2.2e-16; Fig. 3b). These 2,980 candidate covariant SNPs were distributed across all 28 chromosomes, with 39 to 186 SNPs per chromosome (supplementary fig. S8b, Supplementary Material online).

In all the freshwater-resident populations, FWAs of 313 candidate covariant SNPs (10.5%) were fixed, and 1,190 (39.9%) had frequencies higher than 0.8 (Fig. 3c). The average FWA frequency across the 2,980 SNPs was 0.757 in the four freshwater-resident populations, compared to 0.471 in the ancestral Yangtze River Estuary population (Fig. 3c). The average FWA frequency increment across all the candidate covariant SNPs ranged from 0.273 in Hongze Lake to 0.298 in Chaohu Lake (Fig. 3d). Notably, the FWA for 2,970 of the 2,980 covariant SNPs (99.7%) occurred as standing genetic variation in the ancestral Yangtze River Estuary population, indicating that standing genetic variation served as the predominant source for freshwater adaptation in *N. brevirostris*.

### Gene Annotation and Functional Enrichment

**Fig. 4.**
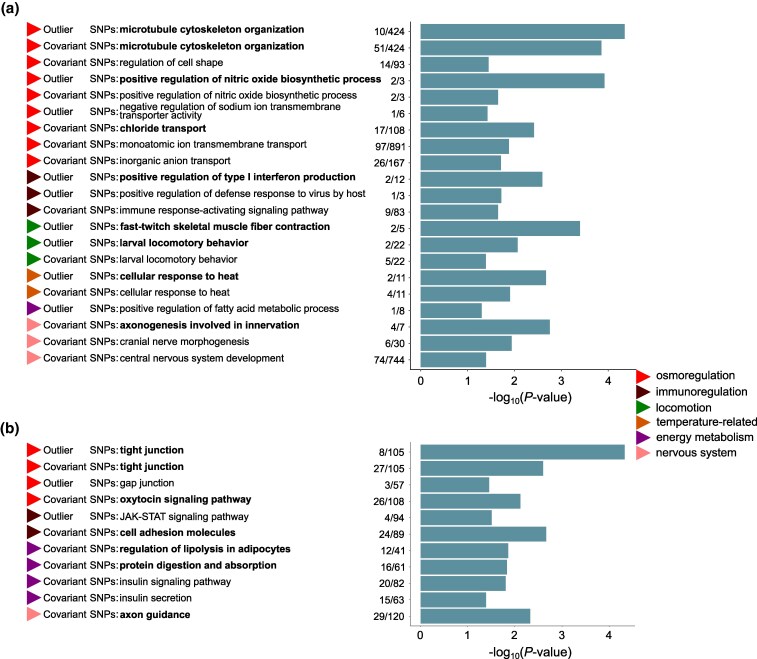
Functional enrichment of genes associated with the 171 candidate outlier SNPs, and the 2,980 candidate covariant SNPs show similar biological functions involved in freshwater adaptation, including GO (a) and KEGG analyses (b). Colored triangles represent broad categories of GO terms or KEGG pathways. Specific terms/pathways and their sources are listed on the left, with bold labels indicating statistically significant results (FDR-BY < 0.1) and non-bold labels representing suggestive associations (FDR-BY ≥ 0.1 but raw *P* < 0.05). Numbers to the left of the bars indicate the associated number and the total number of genes in the corresponding GO term or KEGG pathway. Bar length shows the *P*-values of the GO terms or KEGG pathways on -log_10_ scale. Only the functional terms or pathways potentially related to freshwater adaptation were selected for visualization.

First, annotation of the 171 candidate outlier SNPs identified 123 functional genes (supplementary table S7, Supplementary Material online). Among these, 102 outliers (60%) were located in genic regions, with 55 in exons and 47 in introns. The 55 SNPs in exons were in 29 genes, including eight nonsynonymous variants in seven genes. Some gene categories potentially involved in repeated freshwater adaptation, including (i) ion transporter genes (*ABCB10*, *ABCC10*, and *SLC17A5*) and ion channel genes (*CLIC3*, *KCNAB3_1*, *KCNN4*, and *KCNQ5_2*); (ii) tight junction components (*MARVELD2_4* and *OCLN*); and (iii) cytoskeletal system elements, encompassing tubulin genes (*TUBA1A_3*/*4*/*5*, *TUBB4A_2*/*4*/*5*, and *TUBB5*), actin genes (*ACTA1* and *ACTG1*), and their regulatory genes (*EEF1A1_1*-*4*) (Fig. 2a). Interestingly, five of the eight nonsynonymous mutations occurred in four of these genes (*TUBA1A_4*, *ACTG1*, and *EEF1A1_2*/*3*). Gene Ontology (GO) and Kyoto Encyclopedia of Genes and Genomes (KEGG) analyses, respectively, revealed 16 overrepresented biological process GO terms and 6 significantly enriched KEGG pathways (FDR < 0.1; Fig. 4 and supplementary table S8 to S9, Supplementary Material online), including several potentially correlated with freshwater adaptation, such as microtubule cytoskeleton organization, positive regulation of nitric oxide biosynthetic process, positive regulation of type I interferon production, fast-twitch skeletal muscle fiber contraction, cellular response to heat, and tight junction.

Second, annotation of the 2,980 candidate covariant SNPs identified 1,770 functional genes (supplementary table S10, Supplementary Material online), with 1,717 (58%) located in genic regions (273 in exons, 1,370 in introns, and 74 in UTRs). Among the 273 SNPs in exons, 74 (27%) were nonsynonymous variants. Enrichment analyses revealed functional convergence in freshwater adaptation with the outlier SNP results (Fig. 4), identifying 22 significantly enriched biological process GO terms and 25 KEGG pathways (FDR < 0.1; supplementary tables S11 to S12, Supplementary Material online).

Third, annotations of outlier SNPs detected by both FET and pcadapt in each ecotype pair yielded 493 (Hongze Lake vs. Yangtze River Estuary), 1,373 (Taihu Lake vs. Yangtze River Estuary), 1,373 (Chaohu Lake vs. Yangtze River Estuary), and 252 (Xiangjiang River vs. Yangtze River Estuary) functional genes, respectively (supplementary tables S13 to S16, Supplementary Material online). Although most outlier SNPs were unique to each ecotype pair, both GO and KEGG analyses consistently detected similar enriched biological functions across all ecotype pairs, including osmoregulation, immunoregulation, locomotion, thermal response, and metabolism (supplementary fig. S9 and table S17 to S24, Supplementary Material online), indicating that population-specific genetic adaptation and functional redundancy might also contribute to freshwater adaptation in these freshwater-resident populations.

## Discussion

In this study, we demonstrated that the parallel freshwater adaptation in *N. brevirostris* was characterized by genome-wide parallelism and proceeded through relatively large allele frequency shifts of adaptive standing genetic polymorphisms. These adaptive SNPs were involved in multiple biological functions associated with the polygenic architecture underlying rapid adaptation to freshwater habitats. Our findings highlight the critical role of standing genetic variation in facilitating rapid evolutionary processes. Although classical quantitative genetic theory often emphasizes the predominance of small-effect loci, our results provided evidence that polygenic adaptation also involves genes (loci) with larger effects that contribute to rapid adaptation. These results are consistent with modern quantitative genetic theory that recognizes both large- and small-effect loci as complementary contributors of polygenic adaptation (Pritchard and Di Rienzo 2010; Jain and Stephan 2017; Hayward and Sella 2022).

### Genomic Parallelism Under Parallel Phenotypic Evolution

In evolutionary ecology, one considerable interest is whether parallelism at the phenotypic level is reflected at the genomic level (Elmer and Meyer 2011; Winchell et al. 2023). Our study revealed the shared genetic basis for freshwater adaptation across different freshwater-resident populations, with adaptive SNPs recruited repeatedly from standing genetic variations. The genome-wide signal of parallelism observed is reasonable considering the nature of the founder population in our study; all four freshwater-resident populations were derived from the single ancestral anadromous population. Under stringent criteria, 171 outliers were identified by two outlier detection methods and were shared across at least three freshwater-anadromous ecotype pairs, all showing parallel increments in FWA frequencies in all freshwater-resident populations. The low proportion of shared outliers among the four ecotype pairs can be well understood in the adaptation scenario for polygenic traits, which mostly depends on subtle allele frequency changes across numerous loci (Barghi et al. 2020). Although outlier detection methods have the potential to identify selected loci, they need to work under the precondition of large allele frequency change, which poses challenges in detecting low-impact loci that play a role in freshwater adaptation in all four freshwater-anadromous ecotype pairs (Pinsky et al. 2021).

Our allele frequency covariant analysis reliably confirmed this hypothesis. The four freshwater-resident populations exhibited a large number of SNPs with covariant allele frequency changes, implying that the freshwater adaptation involves many low-impact loci. Among the 5,440 outlier SNPs detected by both FET and pcadapt methods, 3,031 (56%) were also covariant across all the four freshwater-resident populations, although about 680 (12.5%) of them were expected under neutrality, suggesting that about half (43%) of these outliers might have contributed to parallel freshwater adaptation. The other outliers without covariant signals may be the result of genetic redundancy (Barghi et al. 2019), population-specific environmental heterogeneity (Stuart et al. 2017), or false positives due to neutral factors like genetic drift (Naciri and Linder 2020), all of which are common in polygenic adaptation and isolated populations. Under criteria requiring FWA frequency increases ≥ 0.2 in all four freshwater-resident populations, the enrichment results of genes associated with the 2,980 covariant SNPs were highly consistent with those from the 171 candidate outliers. These results further indicated that the number of adaptive SNPs shared among the four freshwater-resident populations could be much larger than that detected by outlier detection methods. However, while we focused on detecting the parallel genetic signals, population-specific adaptive processes could be systematically underestimated. Taken together, while we observed genome-wide parallelism in the freshwater adaptation of *N. brevirostris*, with all four freshwater-resident populations reaching analogous phenotypic endpoints through similar molecular trajectories, population-specific adaptation could also have played a role in the adaptive process.

### Genetic Basis of Freshwater Adaptation

Neutral forces randomly affect the genome, whereas natural selection targets genes participating in specific functions (Roda et al. 2013). Consistent with this perspective, we found that some genes with putative roles in freshwater adaptation were associated with the 171 candidate outlier SNPs. First, proteins associated with cytoskeletal organization play a crucial role in regulating cell volume (Fiol and Kültz 2007), which is essential for tissue homeostasis and cell viability in response to alterations in environmental osmotic conditions (Delpire and Gagnon 2018). Genes related to cytoskeletal compositions, including tubulin genes (Chapman et al. 2021), actin genes (Papakonstanti and Stournaras 2007), and translation elongation factor genes (Novosylna et al. 2015) have been documented to be involved in cell volume regulation. Second, nitric oxide (NO), functioning as a paracrine signal, is implicated in osmotic signaling (Fiol and Kültz 2007) by aiding the adaptive modulation of ion transport (Evans et al. 2004) and regulating chloride cell function (Hyndman et al. 2006). The *DDAH2* gene encodes dimethylarginine dimethylaminohydrolase, which regulates NO generation by controlling cellular methylarginine concentrations (MacAllister et al. 1996). Third, several genes acting as osmotic effectors, including transporter family members (*ABCB10*, *ABCC10*, and *SLC17A5*), exhibit elevated transcription levels under high osmotic conditions (Rasmussen et al. 2019; Jia et al. 2020; Ng et al. 2020). *CLIC3*, a gene encoding a chloride intracellular channel protein, is crucial for maintaining ionic homeostasis (Bossus et al. 2013). Additionally, the genes *KCNAB3_1*, *KCNN4*, and *KCNQ5_2* encode potassium ion voltage-gated channels that regulate intracellular cationic content and cell volume (Parpaite and Coste 2017; Zhao et al. 2023). Fourth, tight junctions (TJs) at intercellular junctions regulate solute flow through the paracellular pathway (Schneeberger and Lynch 2004). Two genes *OCLN* and *MARVELD2_4* correspond to bicellular (bTJ) and tricellular (tTJ) configurations, respectively. Tight junction proteins are widely recognized as key components in osmotic regulations in various fish species, including lamprey (Ferreira-Martins et al. 2021), killifish (Brennan et al. 2018), stickleback (Gibbons et al. 2017), and alewives (Velotta et al. 2017).

All biological functions discussed above are prominently represented in the enrichment analysis. Moreover, we observed other overrepresented GO terms and KEGG pathways related to immunoregulation, locomotion, thermal response, etc., which are also relevant in adaptation to freshwater habitats (Fig. 4). Notably, the results of GO and KEGG enrichment analyses for the genes associated with 2,980 candidate covariant SNPs showed high consistency with the enriched GO terms and KEGG pathways based on the 171 candidate outlier SNPs (Fig. 4). In summary, these candidate SNPs represent key genetic factors contributing to the rapid freshwater adaptation observed in the freshwater-resident populations of *N. brevirostris*.

### Mechanisms of Rapid Adaptation to Freshwater and Frequency Change of FWA

The source of genetic variation that contributes the most to adaptation is a fundamental question in evolutionary biology. The speed at which populations adapt to novel environments depends largely on the availability of adaptive variants (Bomblies and Peichel 2022). Rapid adaptation is expected when standing genetic variation has been pre-tested by selection in a past environment, and a quick frequency shift occurs when selection pressures favor the allele (Barrett and Schluter 2008). Noteworthily, the ancestral anadromous population spends most of their life in the Yangtze River Estuary, where fresh and salt water meet and salinity fluctuates rapidly and continuously, with part of their life history occurring in aquatic environments with salinity below 5‰ (Xu et al. 2018). Consequently, the anadromous population might have been pre-adapted to low salinity, and alleles with higher fitness in low salinity might have been previously selected for. Indeed, the FWA of all 171 candidate outliers and most (99.7%) of the 2,980 candidate covariant SNPs were present in the Yangtze River Estuary population, clearly indicating that the predominant source of adaptive variants was standing genetic variations in the ancestral population. Therefore, adaptation of *N. brevirostris* to freshwater environments can occur rapidly following the settlement of populations in freshwater. Adaptation from standing genetic variations is an important process underlying rapid evolution in natural populations, which has also been detected in other species, such as Songbird (Lai et al. 2019) and *Daphnia* (Chaturvedi et al. 2021).

Complex traits are generally highly polygenic with a large number of loci involved and each contributing a tiny fraction to the overall genetic variance (Goddard et al. 2016). Theoretical expectations of quantitative genetics suggest that most of these infinitesimal sites will experience only subtle changes in allele frequency for the evolution of polygenic adaptive traits (Pritchard and Di Rienzo 2010). However, the average FWA frequency increment of the 171 candidate outliers exceeded 0.36, with most FWA fixed. A total of 2,980 covariant SNPs were found with ≥ 0.2 FWA frequency increment across all freshwater-resident populations, with 10.5% of FWA fixed and 39.9% of FWA frequencies higher than 0.8. Theoretical simulations indicate that the probability of allele fixation increases with the magnitude of the beneficial effect of the selectively favored alleles (Hermisson and Pennings 2005). As mentioned above, the ancestral anadromous population might have been pre-adapted to low salinity, and the FWA might have a large beneficial effect when populations were enclosed in freshwater habitats. Transitions from marine to freshwater environments represent dramatic shifts between “adaptive zones” (Lee and Bell 1999), and this large optimum shift could also exert strong selective pressure, leading to the increased frequency of the FWA with a large beneficial effect. Compared with adaptation from new mutations, adaptation from standing genetic variations is also likely to lead to the fixation of more alleles with small effect (Hermisson and Pennings 2005; Barrett and Schluter 2008). Furthermore, in adaptation from standing genetic variation to environmental change, the fixation probability of the successful allele in a new environment is only weakly dependent on the selection coefficient when the initial frequency of the successful allele is high (Hermisson and Pennings 2005). In the Yangtze River Estuary population, the average FWA frequency of the 171 candidate outliers was notably high at 0.621, and the average FWA frequency for the 2,980 candidate covariant SNPs was 0.471. As a consequence, the high initial frequency of FWA present as standing variation in the anadromous population might have reduced the average fixation time and increased the fixation probability, resulting in the fixation and high frequency increment of FWA in the freshwater-resident populations. These results indicated that the rapid adaptive evolution of polygenic traits in response to environmental changes could proceed via relatively large allele frequency change at many adaptive loci, given that their initial standing frequencies in the ancestral population are high.

## Conclusion

Understanding how natural populations adapt to rapid changes in the environment is a fundamental focus of ecological and evolutionary biology. Repeated evolutionary events provide a powerful means for addressing genetic mechanisms underlying rapid evolution. Here, we performed such an investigation in a Salangid icefish (*N. brevirostris*) by focusing on the genetic architecture underlying the adaptation of populations to freshwater after their recent isolation in lakes from the ancestral anadromous population. We demonstrated that the parallel adaptation to freshwater in *N. brevirostris* was characterized by genome-wide parallelism. We found that parallel genome-wide selection of standing genetic variations across a highly polygenic genetic architecture made a considerable contribution to the rapid freshwater adaptation, which was underlain by relatively large frequency changes of many adaptive alleles. This work provided new insights into the mechanisms by which natural populations evolve to abrupt changes in the environment and highlights the importance of standing genetic variation for the adaptive capacity of populations facing environmental changes.

## Materials and Methods

### Sampling, Library Preparation, and Sequencing

A total of 161 specimens of *N. brevirostris* were collected from five populations during 2018 to 2023, with 20 individuals from the Yangtze River Estuary, 46 individuals from Hongze Lake, 48 individuals from Taihu Lake, 24 individuals from Chaohu Lake, and 23 individuals from Xiangjiang River. The Yangtze River Estuary specimens were referred to as “ancestral anadromous population”. Samples from Hongze Lake, Taihu Lake, Chaohu Lake, and Xiangjiang River were referred to as “derived freshwater-resident populations”. Muscle tissues were kept individually in 95% ethanol and stored at −80°C. Genomic DNA were extracted following the standard phenol-chloroform extraction method. DNA extracts were visualized on 1% agarose gels to assess quality and were subsequently quantified using Qubit fluorometer. Whole genome sequencing libraries with insert size ∼350 bp were constructed and then sequenced on the DNBSEQ-T7 platform using 150-bp paired-end sequencing with a minimum coverage of ∼20× for each individual.

### Data Filtering, SNP Genotyping, and Filtering

For raw reads, adapters and low-quality reads were removed using FASTP v0.23.2 (Chen et al. 2018) with default parameters. The generated clean reads were mapped to the chromosome-level genome of *N. brevirostris* (GenBank accession: GCA_044705665.1) using BWA-MEM v0.7.17 (Li 2013) with default parameters. Aligned BAM files were sorted using Sambamba v1.0.1 (Tarasov et al. 2015), and PCR duplicates were marked using Samblaster v0.1.26 (Faust and Hall 2014). SNP calling was performed by BCFtools v1.10.2 in SAMtools v1.10 (Li et al. 2009) based on a Bayesian framework, and SNPs were stored in a VCF file. To retain high-quality SNPs for the downstream analysis, we used the following criteria to filter all SNPs identified: (i) only biallelic SNPs were retained; (ii) SNP overall quality score (*Q*) ≥ 30 and genotype quality (*GQ*) ≥ 20; (iii) minimum coverage depth for each individual of each SNP site ≥ 7; (iv) SNP were called in at least 90% individuals overall and 12 individuals for each population; (v) global observed heterozygosity (*H*_O_) ≤ 0.8; (vi) global MAF ≥ 0.05, and (vii) retain the SNP with local MAF ≥ 0.2 in any of the five populations but failed to meet the criteria of the global MAF ≥ 0.05. SNP VCF file was converted into other formats using PLINK v1.90b6.21 (Purcell et al. 2007) or an in-house script.

For population genetic structure and evolutionary history analysis across all populations, we generated a subset of neutral and unlinked SNPs. First, any SNPs that were identified as outliers by either of the two detection methods in any freshwater-anadromous ecotype pair were removed (Materials and Methods); second, only one SNP per 10-kb region was kept to remove putative LD using VCFtools v0.1.15 (Danecek et al. 2011).

### Genetic Diversity and Population Genetic Structure

ANGSD v0.941 (Korneliussen et al. 2014) was employed to estimate individual-level heterozygosity, defined as the proportion of heterozygous sites per individual. The following parameters were applied: “-C 50 -setMinDepthInd 5 -minQ 20 -uniqueOnly 1 -minMapQ 20.”

Population genetic structure was characterized using three approaches. First, the model-based program Admixture v1.3.0 (Alexander et al. 2009) was used to determine population structure. We applied *K* values ranging from 1 to 6 with ten replicates per *K* and determined the optimal *K* using StructureSelector (Li and Liu 2018). Second, PCA was implemented using PLINK, and results were visualized with ggplot2 v3.5.0 R package (Ginestet 2011). Third, an individual-based NJ tree was constructed using VCF2DiS v1.53 (Xu et al. 2025) based on the whole SNP dataset (VCF format), and visualized in iTOL v6 (Letunic and Bork 2024). The node support values were assessed using the bootstrapping algorithm with 1,000 replicates. Meanwhile, the pairwise *F*_ST_ values among all populations were calculated in Arlequin v3.5.2.2 (Excoffier and Lischer 2010), with statistical significance assessed through 10,000 permutations followed by Bonferroni correction for multiple testing.

### Evolutionary History and Recent Demographic Dynamics

To reconstruct the evolutionary trajectories of how the four freshwater-resident populations derived from their common anadromous ancestor, we conducted demographic inference using the Approximate Bayesian Computation (ABC) approach as implemented in DIYABC-RF v1.2.1 (Collin et al. 2021). Given the complexity of evaluating all possible scenarios simultaneously, we focused on the nine most probable scenarios for analysis (supplementary fig. S10, Supplementary Material online). For all simulations, a subset of 20,000 SNPs was randomly extracted from the neutral unlinked dataset mentioned above. Population sizes were assigned broad priors with a uniform distribution (10 to 100,000 for each parameter), while divergence timeframes were assigned narrow priors with a uniform distribution (10 to 100 for each parameter), accounting for the true demographic history of the populations. Following DIYABC-RF documentation, each simulation was performed with 1 × 10^5^ runs, totaling 9 × 10^5^ runs across all scenarios. After generating the training set simulations, the “Random Forest analyses” module was employed for model scenario choice and parameter estimation based on 1,000 random forest trees.

The historical *N*_e_ for the five populations was estimated using the LD method in GONE software (Santiago et al. 2020). The following settings were applied, including unknown phase, Haldane's correction for genetic distances, cMMb = 1, no MAF filtering, and use of all SNPs, even those with missing data. A randomly selected set of 50,000 SNPs per chromosome was used for each of the 20 replicates. The maximum recombination rate was set to *c* = 0.05, in accordance with the default value. The estimates were made without knowledge of the genetic map, assuming a uniform recombination rate across the genome. The geometric mean of the *N*_e_ estimates from the 20 replicates was calculated for each population. Previous GONE applications to Baltic herring (Atmore et al. 2022), turbot and gilthead seabream (Saura et al. 2021) showed that the *N*_e_ of the four most recent generations were considered pseudo-products of the calculation; we thus removed these results from the analysis.

Genetic drift is a random process that can alter allele frequencies in populations, with its effects being particularly pronounced in small populations. Theoretical predictions suggest that strong genetic drift typically produces a uniform allele frequency distribution resulting from stochastic loss of rare variants, and weak genetic drift generally exhibits a pronounced L-shaped SFS characterized by abundant rare alleles. Meanwhile, we employed Tajima's *D* as a complementary statistic to validate demographic inferences obtained from GONE. At the genome-wide level, positive Tajima's *D* values indicate an excess of intermediate-frequency alleles consistent with population contraction, while negative values reflect an abundance of rare alleles suggestive of recent expansion. Therefore, we performed two complementary analyses to evaluate these dynamics: (i) calculated genome-wide MAF for each of the five populations using PLINK while deliberately avoiding any MAF filtering to capture the complete spectrum of allele frequencies; (ii) computed genome-wide Tajima's *D* values for each population using 100-kb sliding windows (with zero step size) in VCFtools.

### Detection of Outlier SNPs

Outlier SNPs potentially associated with adaptive divergences between the anadromous and freshwater-resident ecotypes were identified by two methods. First, we applied FET to examine allele frequency differences across the whole SNP dataset. SNPs displaying statistical significance (FDR < 0.05) in FET were designated as outliers. Second, we implemented the pcadapt v4.3.5 R package to detect outliers (Privé et al. 2020). Pcadapt assumes that candidate SNPs are outliers concerning how they are related to population structure based on PCA, which can handle admixed individuals and does not require grouping individuals into populations. We set *K* = 2 to reflect population structure, and any SNP with a *Q*-value < 0.1 was deemed an outlier. To minimize false positives, we applied stringent filtering criteria: (i) only SNPs consistently identified as outliers by both FET and pcadapt methods for each freshwater-anadromous ecotype pair were retained, (ii) among these, only outliers shared across at least three ecotype pairs were kept, and (iii) from this subset, only those exhibiting consistent allele frequency change directions across all the four ecotype pairs were considered as candidate outlier SNPs contributing to the parallel freshwater adaptation. Subsequently, to statistically evaluate the significance of outlier SNP overlaps across all combinations of the four freshwater-anadromous ecotype pairs, we performed hypergeometric tests using the SuperExactTest v1.1.0 R package (Wang et al. 2015). The number of SNPs in the whole SNP dataset served as the background set. Finally, we calculated the FWA frequency for candidate outlier SNPs in the ancestral anadromous population and the derived freshwater-resident populations, as well as their shifts in the four ecotype pairs.

### Identification of Covariant SNPs

To test whether there are more parallel signals than that identified by the outlier detection methods, we conducted allele frequency covariant analysis through the following steps: (i) calculating the population-level allele frequency at each site across the whole dataset, (ii) extracting SNPs that meeting the FWA definition using an in-house script, and (iii) defining the candidate covariant SNPs as those exhibiting FWA frequency increments ≥ 0.2 to minimize the effects of random genetic drift and sampling variance.

To assess the neutral expectation for covariant SNP distributions and statistically evaluate whether the observed results reflected natural selection rather than chance effects, we carried out forward simulations using SLiM v4.3 (Haller and Messer 2023). The simulated genome was configured with: (i) neutral selection model across the whole genome, (ii) uniform recombination rate of 1e-8 per site per generation, and (iii) initial genetic variation derived from the ancestral Yangtze River Estuary population (VCF-based initialization). The *N*_e_ estimated by GONE were parameterized, with each of the five simulated populations assigned their corresponding average *N*_e_ values calculated across 5 to 70 generations. To ensure computational feasibility, any mean *N*_e_ value exceeding 100,000 was truncated to 100,000 for subsequent analyses. All simulations spanned 70 generations to match the actual demographic history. To account for potential variance, we performed 200 independent simulations and then 10 random samplings for each independent simulation, totaling 2,000 simulation-sampling replicates. The sampling sizes of each random sampling were the same as the empirical sample size of our study populations. Following these simulations, we established the neutral expectation by summarizing the distribution of covariant SNP counts with FWA frequency increments ≥ 0.2, and performed *Z*-tests to statistically compare our observed number against this neutral distribution. The FWA frequencies at these SNPs were visualized using the pheatmap v1.0.12 R package (Kolde 2019) with the clustering type using Minkowski distance.

### Gene Annotation and Enrichment Analyses

Variants were annotated using snpEFF v5.2c (Cingolani et al. 2012) based on the genome annotation file (accession link: https://figshare.com/s/c056fdfb62dbeab041f9). The snpEFF assigned properties such as gene names and consequences (e.g. missense, synonymous, etc.) to each SNP. Upstream and downstream variants were defined as SNPs located within 5-kb in physical distance from a gene.

Two methods were selected for enrichment analysis to check whether any biological functions were enriched. One is GO enrichment analysis, we first aligned the protein sequences of *N. brevirostris* to the protein database of *Danio rerio*, and then the related GO terms of each gene were obtained from the Gene Ontology Annotation (GOA) database (accession link: http://ftp.ebi.ac.uk/pub/databases/GO/goa/). Enrichment analysis for GO terms was performed by the topGO v2.52.0 R package (Alexa and Rahnenfuhrer 2016) using the “weight01” algorithms. The other is KEGG pathway enrichment analysis. We initially uploaded the protein sequences to eggNOG-mapper and KEGG BlastKOALA for functional annotation, acquiring the KO ID for each gene, and then, the results from both tools underwent manual optimization. Enrichment analysis for KEGG pathway was conducted using hypergeometric tests with the clusterProfiler v4.8.3 R package (Wu et al. 2021). Statistically significant enrichment results were defined as GO terms and KEGG pathways meeting FDR-BY < 0.1. To capture additional biological signals, we also retained terms and pathways showing suggestive significance (raw *P* < 0.05 despite FDR-BY ≥ 0.1).

## Supplementary Material

msaf160_Supplementary_Data

## Data Availability

The sequencing data that support the findings of this study are openly available in the NCBI Sequence Read Archive (SRA) under BioProject accession No. PRJNA1140889. All the customed codes used in this study are available at https://github.com/lyl8086/NbrAdaptation, last accessed June 23, 2025.
