# Supplementary material for: Genome-wide Parallelism Underlies Rapid Freshwater Adaptation Fueled by Standing Genetic Variation in a Wild Fish
Source: Mol Biol Evol. 2025 Jul 3;42(7):msaf160. doi: 10.1093/molbev/msaf160 (PMC12279437; doi:10.1093/molbev/msaf160)
Supplement: msaf160_Supplementary_Data [file msaf160_supplementary_data.zip › msaf160_Supplementary_File 1.pdf]

Supplementary Materials for

Genome-wide Parallelism Underlies Rapid Freshwater Adaptation Fueled by Standing Genetic Variation in a Wild Fish

Hao Yang[1,2,3,†], Yu-Long Li[1,2,†], Teng-Fei Xing[1,2], Jian-Hui Wu[4,5], Ting Wang[4,5], Ming-Sheng Zhu[6], and Jin-Xian Liu[1,2,*]

[1] Key Laboratory of Marine Ecology and Environmental Sciences, Institute of Oceanology, Chinese Academy of Sciences, Qingdao 266071, China

[2] Laboratory for Marine Ecology and Environmental Science, Qingdao Marine Science and Technology Center, Qingdao 266237, China

[3] University of Chinese Academy of Sciences, Beijing 100049, China

[4] Shanghai Aquatic Wildlife Conservation and Research Center, Shanghai 200092, China

[5] Shanghai Monitoring Station of Aquatic Biological Resources in the Yangtze River Basin, Shanghai 202162, China

[6] Office of Taihu Fishery Management Committee, Suzhou 215104, China

[†] These authors contribute equally to this work.

[*] **Corresponding author:** E-mail: jinxianliu@gmail.com.

This PDF file includes:

Figures S1 to S10

Tables S1 to S24 (See attached Excel file)

Contents

Supplementary figures

Fig. S1 The main view and top view of *Neosalanx brevirostris*.

Fig. S2 Density plot of SNPs identified on the 28 chromosomes of the *Neosalanx brevirostris* genome.

Fig. S3 An individual-level neighbor-joining tree with branch length information.

Fig. S4 CLUMPAK major mode plots for the Admixture results based on neutral and unlinked SNPs.

Fig. S5 PCA showing the clustering of individuals along the first and third PCs, and the second and third PCs.

Fig. S6 Recent demographic dynamics of the five populations estimated using GONE.

Fig. S7 Distribution of the minor allele frequency of SNPs for each population.

Fig. S8 Distribution of the 171 candidate outlier SNPs and the 2,980 candidate covariant SNPs on chromosomes of the genome.

Fig. S9 Consistent patterns of biological functional enrichment related to the freshwater adaptation were observed across all four freshwater-anadromous ecotype pairs.

Fig. S10 Nine possible scenarios simulated in DIYABC-RF to reconstruct the evolutionary history of *Neosalanx brevirostris* populations.

Supplementary tables (See attached Excel file)

Table S1 Site locations, date of collection, sample size, individual heterozygosity and coverage depth for all samples included in this study.

Table S2 The results of classification vote for nine evolutionary history scenarios and estimations for the posterior probability of the best supported scenario.

Table S3 Genome-wide Tajima's *D* statistics (10-kb windows, non-overlapping) for five populations.

Table S4 Numbers of outlier SNPs detected by Fisher's exact test and pcadapt for the four anadromous-freshwater ecotype pairs.

Table S5 The observed and expected overlap of the shared outlier SNPs and their statistical significance across all combinations of the four freshwater-anadromous ecotype pairs.

Table S6 Freshwater-favored allele (FWA) frequency for the 2,980 candidate covariant SNPs in the five populations analyzed.

Table S7 Gene annotations of the 171 candidate outlier SNPs.

Table S8 Gene ontology (GO) enrichment of biological process for genes annotated from the 171 candidate outlier SNPs.

Table S9 Kyoto Encyclopedia of Genes and Genomes (KEGG) enrichment for genes annotated from the 171 candidate outlier SNPs.

Table S10 Gene annotations of the 2,980 candidate covariant SNPs.

Table S11 Gene ontology (GO) enrichment of biological process for genes annotated from the 2,980 candidate covariant SNPs.

Table S12 Kyoto Encyclopedia of Genes and Genomes (KEGG) enrichment for genes annotated from the 2,980 candidate covariant SNPs.

Table S13 Gene annotations of outlier SNPs detected by both FET and pcadapt methods in Hongze Lake verse Yangtze River Estuary pair.

Table S14 Gene annotations of outlier SNPs detected by both FET and pcadapt methods in Taihu Lake verse Yangtze River Estuary pair.

Table S15 Gene annotations of outlier SNPs detected by both FET and pcadapt methods in Chaohu Lake verse Yangtze River Estuary pair.

Table S16 Gene annotations of outlier SNPs detected by both FET and pcadapt methods in Xiangjiang River verse Yangtze River Estuary pair.

Table S17 Gene ontology (GO) enrichment of biological process for genes from Hongze Lake verse Yangtze River Estuary pair.

Table S18 Gene ontology (GO) enrichment of biological process for genes from Taihu Lake verse Yangtze River Estuary pair.

Table S19 Gene ontology (GO) enrichment of biological process for genes from Chaohu Lake verse Yangtze River Estuary pair.

Table S20 Gene ontology (GO) enrichment of biological process for genes from Xiangjiang River verse Yangtze River Estuary pair.

Table S21 Kyoto Encyclopedia of Genes and Genomes (KEGG) enrichment for genes from Hongze Lake verse Yangtze River Estuary pair.

Table S22 Kyoto Encyclopedia of Genes and Genomes (KEGG) enrichment for genes from Taihu Lake verse Yangtze River Estuary pair.

Table S23 Kyoto Encyclopedia of Genes and Genomes (KEGG) enrichment for genes from Chaohu Lake verse Yangtze River Estuary pair.

Table S24 Kyoto Encyclopedia of Genes and Genomes (KEGG) enrichment for genes from Xiangjiang River verse Yangtze River Estuary pair.

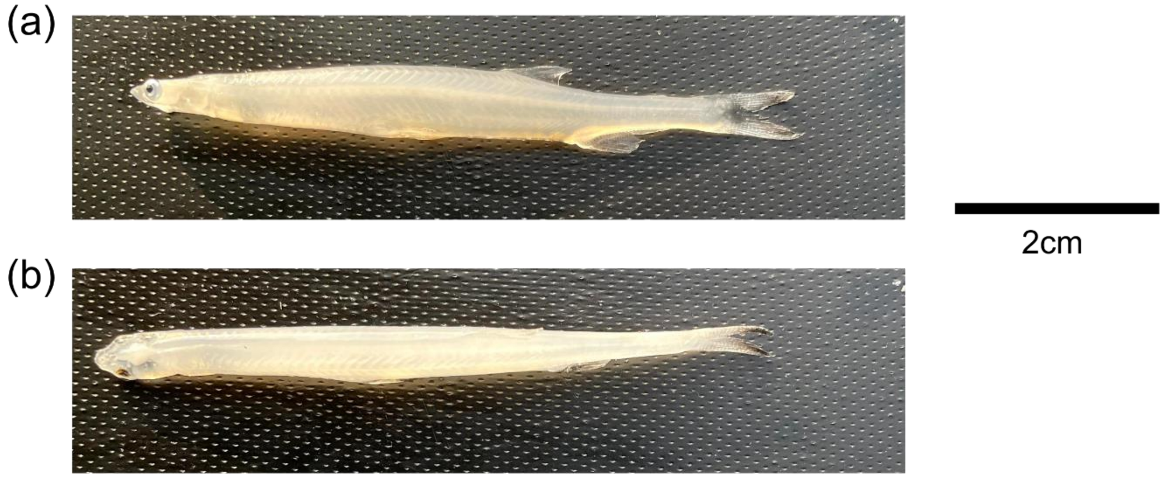

Fig. S1 The main view (a) and top view (b) of *Neosalanx brevirostris*.

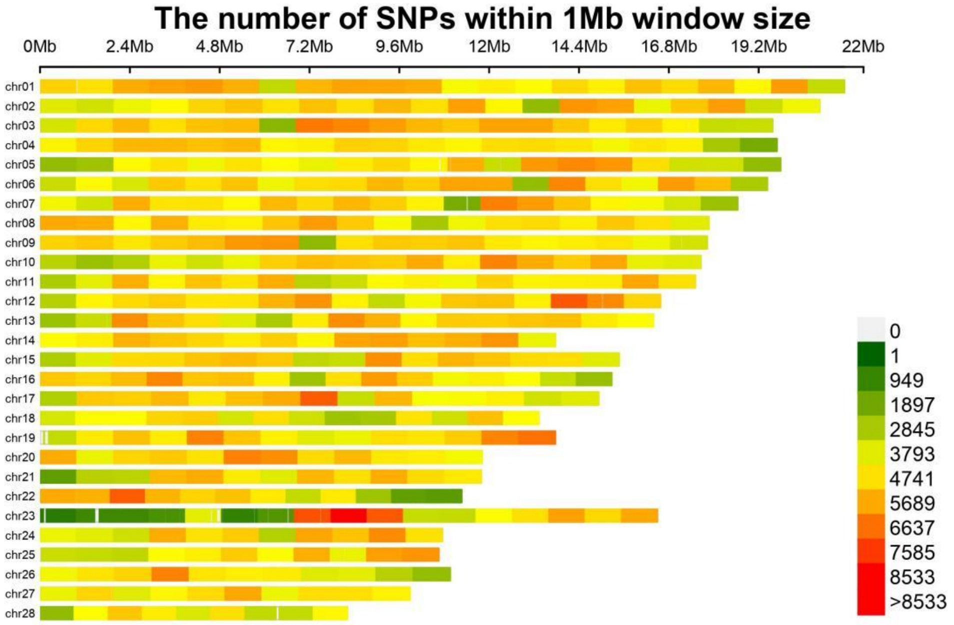

Fig. S2 Density plot of SNPs identified on the 28 chromosomes of the *Neosalanx brevirostris* genome.

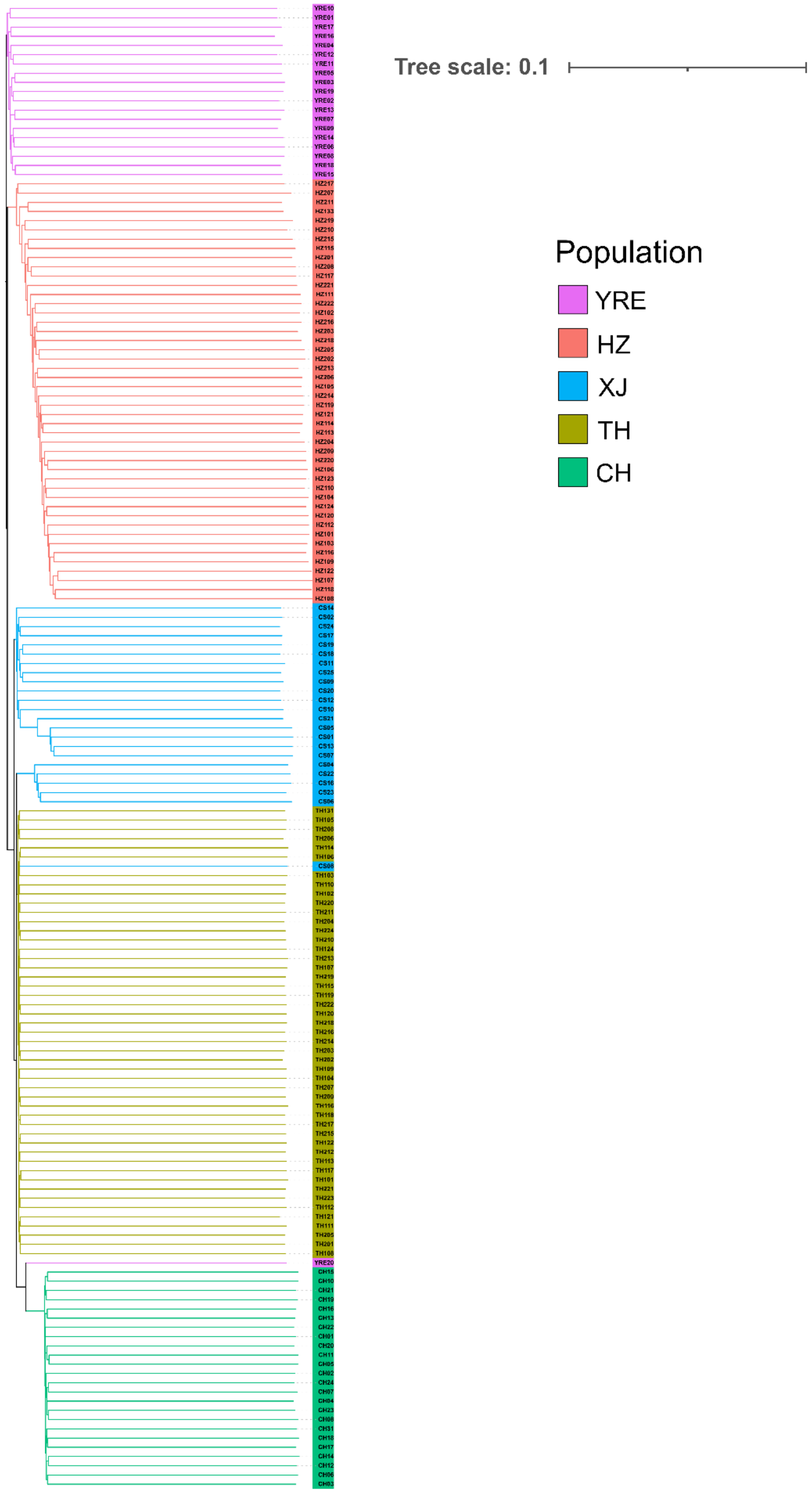

Fig. S3 An individual-level neighbor-joining (NJ) tree with branch length information. HZ, Hongze Lake; TH, Taihu Lake; CH, Chaohu Lake; XJ, Xiangjiang River; YRE, Yangtze River Estuary.

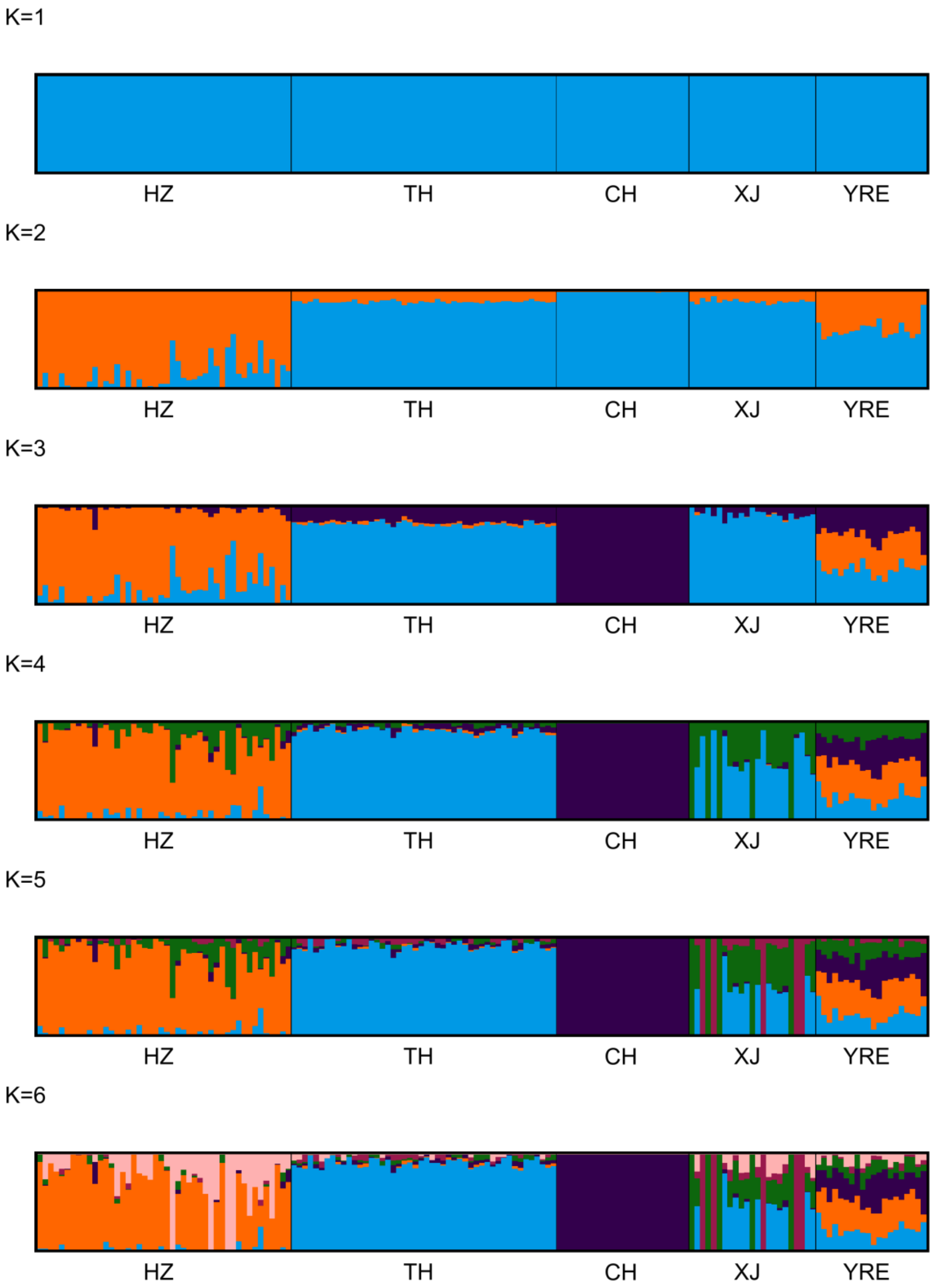

Fig. S4 CLUMPAK major mode plots for the Admixture results (*K* = 1 to 6) based on neutral and unlinked SNPs. HZ, Hongze Lake; TH, Taihu Lake; CH, Chaohu Lake; XJ, Xiangjiang River; YRE, Yangtze River Estuary.

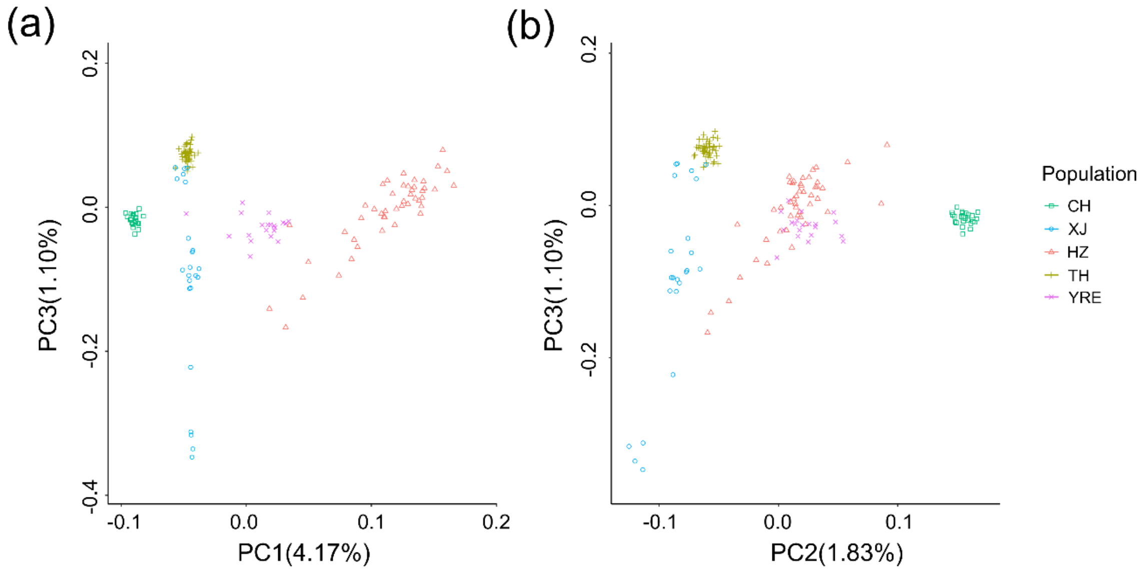

Fig. S5 PCA showing the clustering of individuals along the first and third PCs (a), and the second and third PCs (b). The color and shape label corresponds to different populations. HZ, Hongze Lake; TH, Taihu Lake; CH, Chaohu Lake; XJ, Xiangjiang River; YRE, Yangtze River.

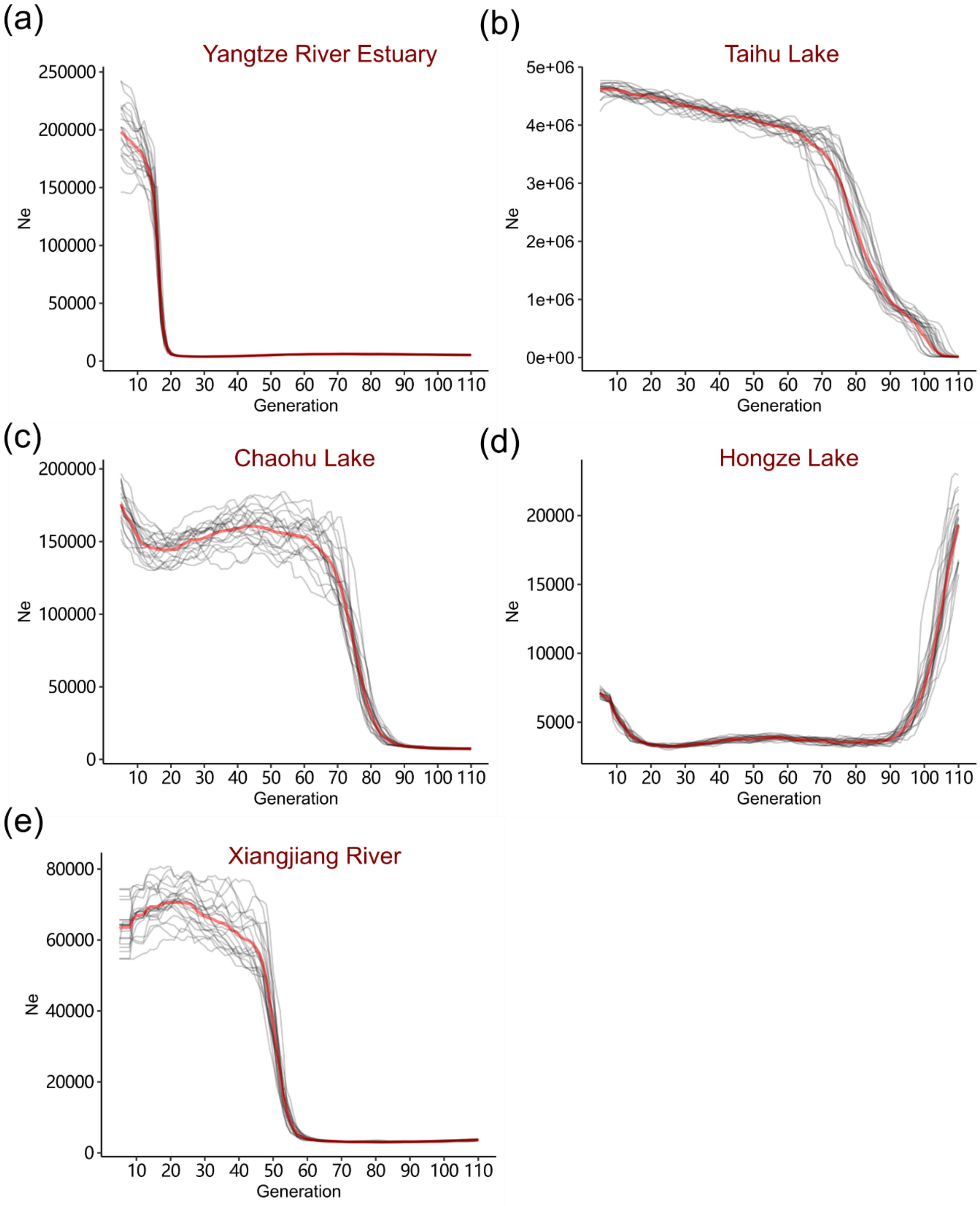

Fig. S6 Recent demographic dynamics of the five populations estimated using GONE, excluding the four most recent generations. Gray lines represent individual replicate estimates (n=20), while the red line shows their geometric mean. All analyses were performed with a maximum recombination frequency threshold of $c = 0.05$.

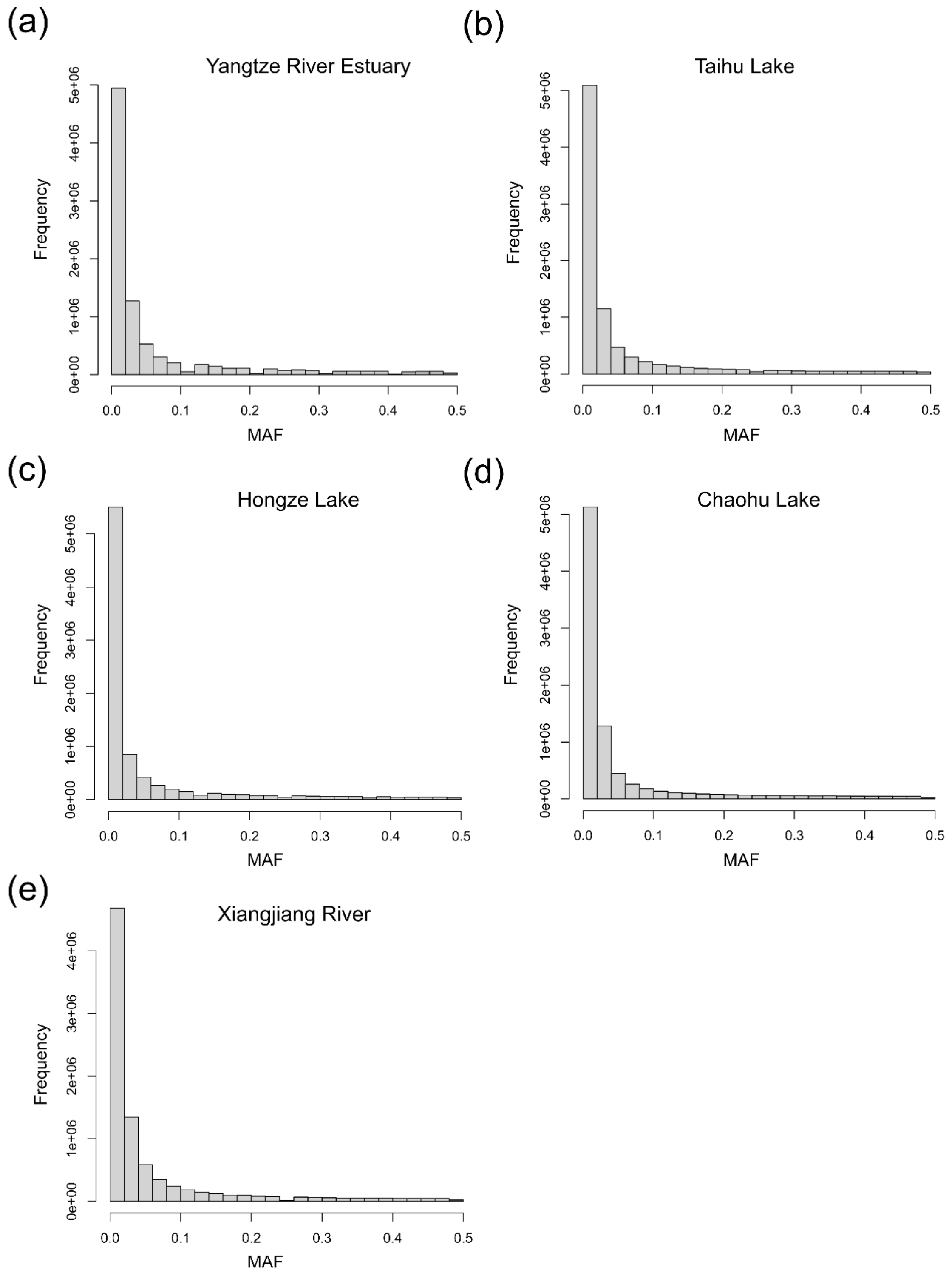

Fig. S7 Distribution of the minor allele frequency (MAF) of SNPs for each population.

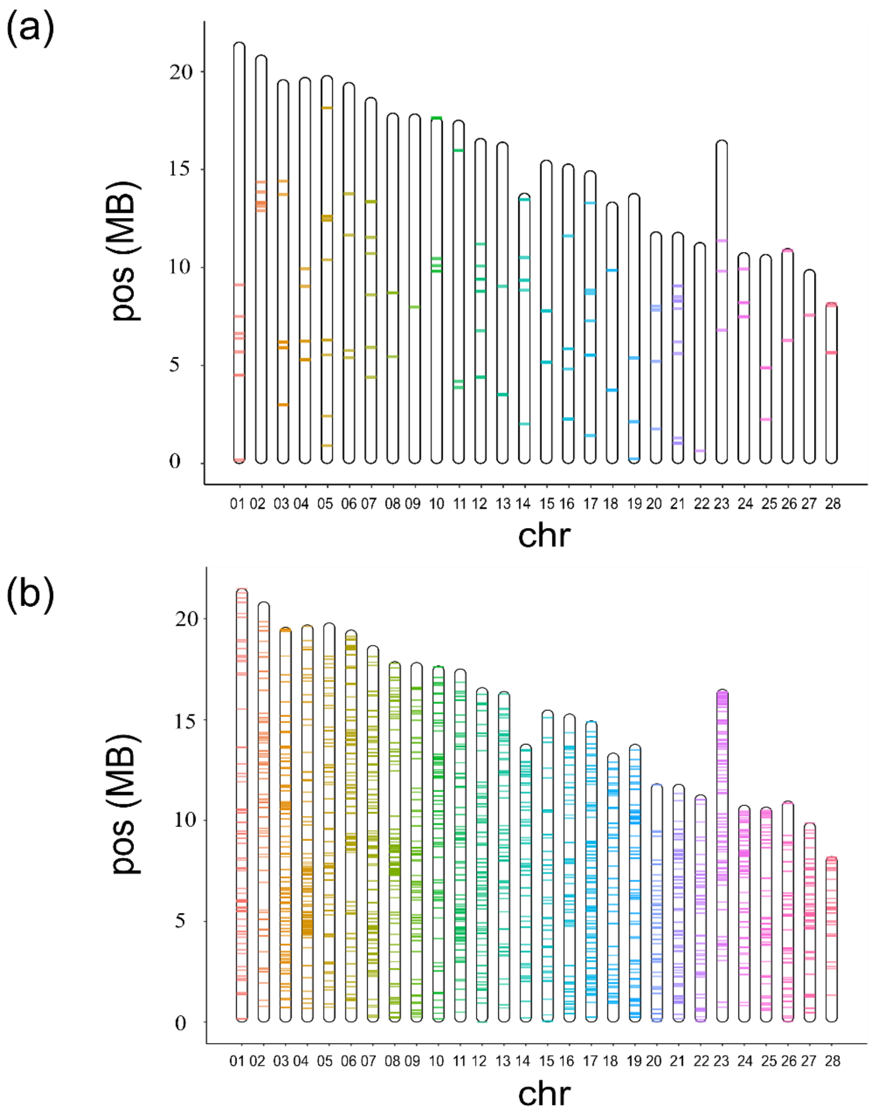

Fig. S8 Distribution of the 171 candidate outlier SNPs (a) and the 2,980 candidate covariant SNPs (b) on chromosomes of the genome.

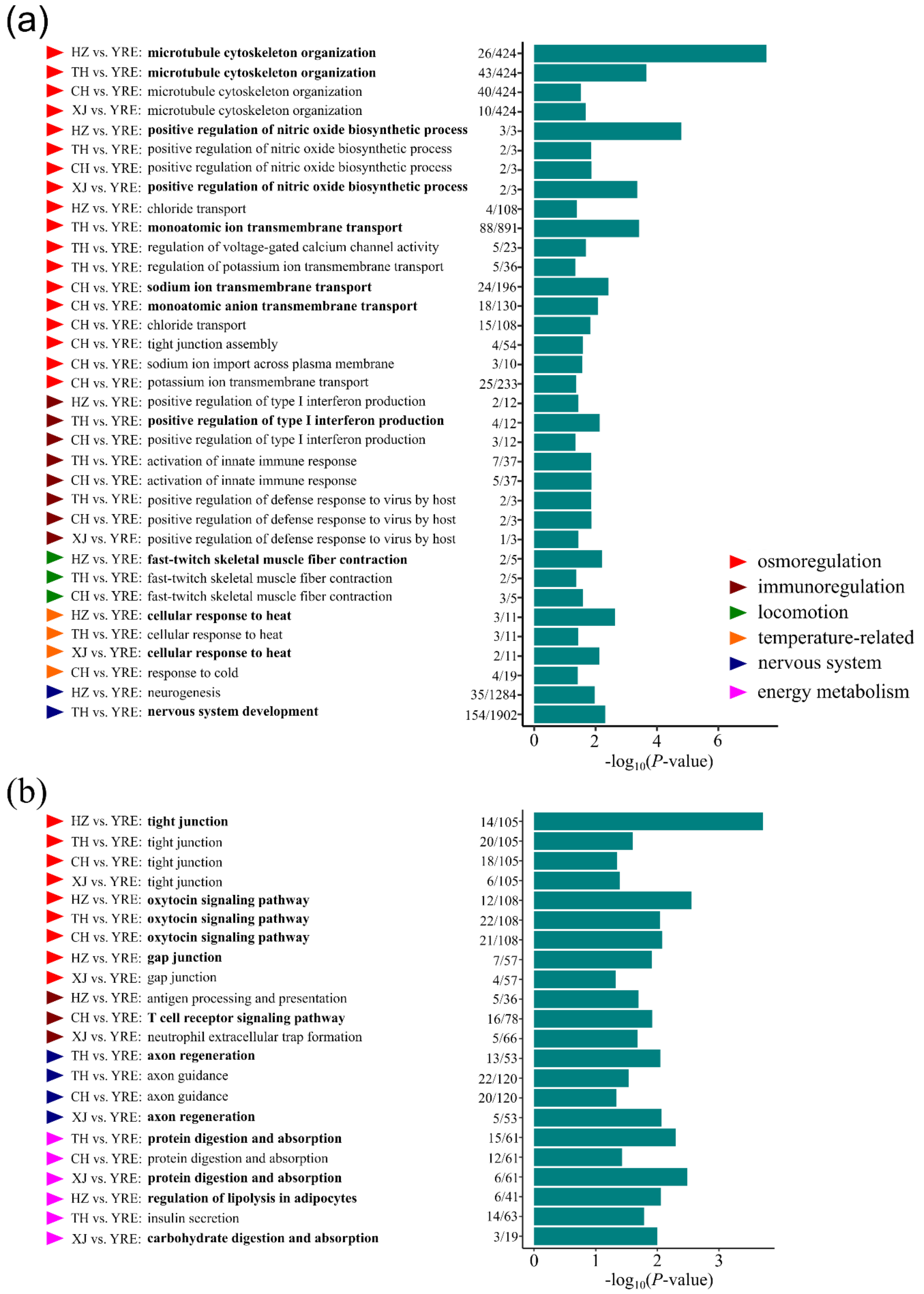

Fig. S9 Consistent patterns of biological functional enrichment related to the freshwater adaptation were observed across all four freshwater-anadromous ecotype pairs, including both

GO (a) and KEGG (b) analyses. Colored triangles represent broad categories of GO terms or KEGG pathways. Specific terms/pathways and their sources are listed on the left, with bold labels indicating statistically significant results (FDR-BY < 0.1) and non-bold labels representing suggestive associations (FDR-BY ≥ 0.1 but raw $P < 0.05$). Numbers to the left of the bars indicate the associated number and the total number of genes in the corresponding GO term or KEGG pathway. Bars length shows the *P*-values of the GO terms or KEGG pathways in -log10 scale. Only the functional terms or pathways potentially related to freshwater adaptation were selected for visualization. HZ, Hongze Lake; TH, Taihu Lake; CH, Chaohu Lake; XJ, Xiangjiang River; YRE, Yangtze River Estuary.

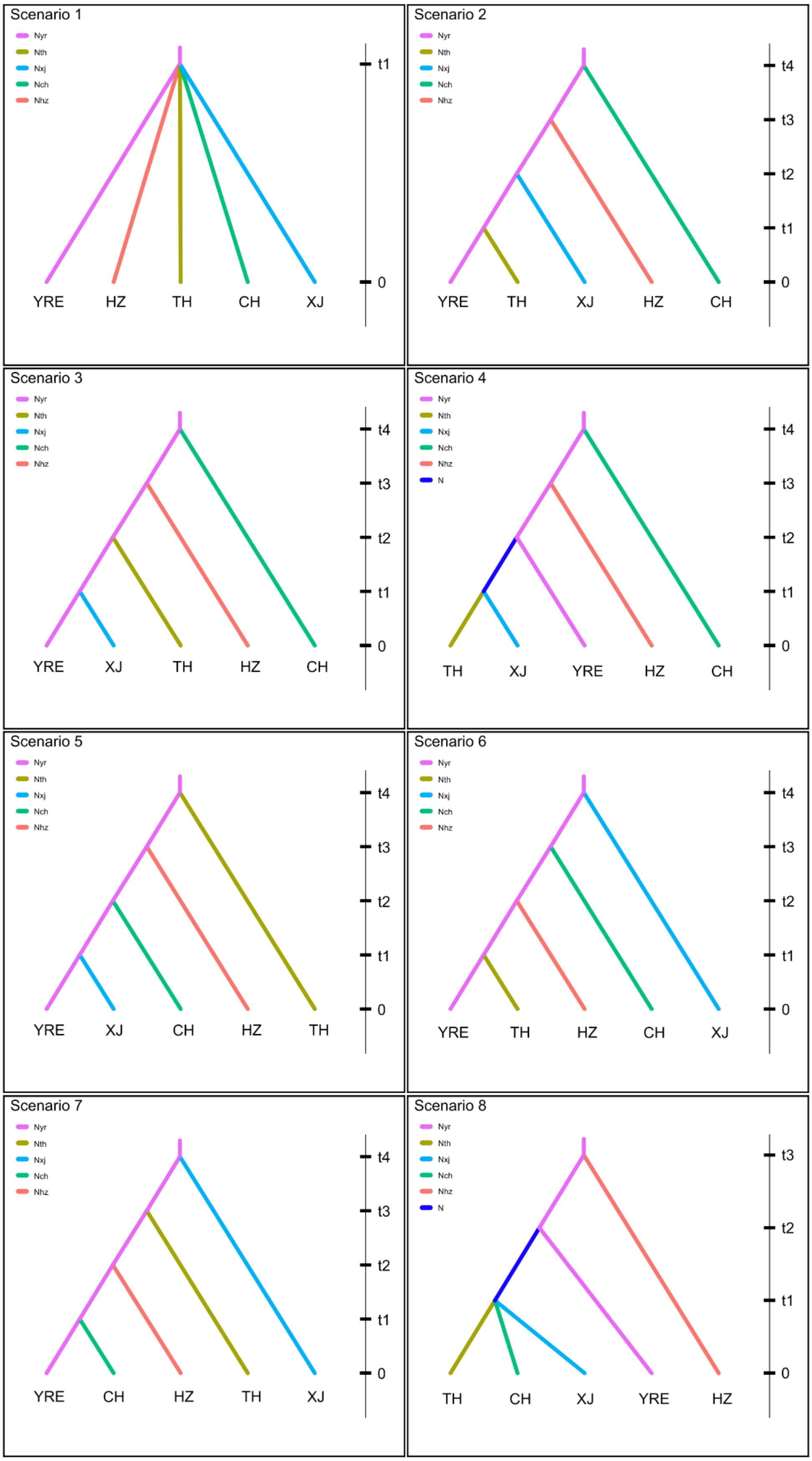
Scenario 1
Nyr
Nth
Nxj
Nch
Nhz
t1
0
YRE HZ TH CH XJ
Scenario 2
t4
t3
t2
t1
0
YRE TH XJ HZ CH
Scenario 3
YRE XJ TH HZ CH
Scenario 4
N
TH XJ YRE HZ CH
Scenario 5
YRE XJ CH HZ TH
Scenario 6
YRE TH HZ CH XJ
Scenario 7
YRE CH HZ TH XJ
Scenario 8
TH CH XJ YRE HZ

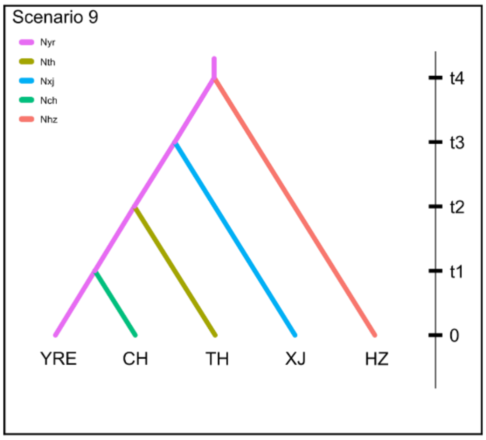

Fig. S10 Nine possible scenarios simulated in DIYABC-RF to reconstruct the evolutionary history of *Neosalanx brevirostris* populations. In these scenarios, t# represents the time-scale in terms of the number of generations (t1, t2, t3, and t4 respectively represent four assumed differentiation time points in the scenarios, satisfying t4 ≥ t3 ≥ t2 ≥ t1), and N represents the effective population size (N_e) of an unknown population. Nyr: N_e of Yangtze River Estuary; Nhz: N_e of Hongze Lake; Nth: N_e of Taihu Lake; Nch: N_e of Chaohu Lake; Nxj: N_e of Xiangjiang River. YRE, Yangtze River Estuary; HZ, Hongze Lake; TH, Taihu Lake; CH, Chaohu Lake; XJ, Xiangjiang River.

Scenario 1 represents the simplest model, where the four freshwater-resident populations are derived from their ancestral population simultaneously.

Scenario 2, 3 and 4 are based on the genetic distances between the four freshwater-resident populations and their ancestral population. In view of the small genetic divergence observed in Taihu Lake and Xiangjiang River population, we developed three distinct models.

Scenario 5 and 6 are based on geographic proximity and are divided into two models: one considering populations from distant to close locations from the Yangtze River Estuary population, and the other considering populations from close to distant locations.

Scenario 7 takes into account the historical background of the lakes, where lakes that were formed earlier are assumed to have given rise to their freshwater-resident populations at an earlier time.

Scenario 8 focuses on the relationship between the lakes and the Yangtze River basin. Hongze Lake, located within the Huai River basin, which is a tributary of the Yangtze River, while the other three lakes are situated along the main course of the Yangtze River.

Scenario 9 is based on the topological structure of individual-based neighbor-joining (NJ) phylogenetic relationships.
